# Activation of Alkaline Irrigation Fluids in Endodontics

**DOI:** 10.3390/ma10101214

**Published:** 2017-10-23

**Authors:** Laurence J. Walsh, Roy George

**Affiliations:** 1 The University of Queensland School of Dentistry, Herston, Brisbane QLD 4006, Australia; 2 Griffith University School of Dentistry and Oral Health, Southport QLD 4215, Australia; drroygeorge@gmail.com

**Keywords:** endodontics, irrigation, EDTA, sodium hypochlorite, ultrasonics, laser activation, fluid extrusion, smear layer

## Abstract

In conventional endodontic treatment, alkaline solutions of sodium hypochlorite (NaOCl) and ethylenediaminetetraacetic acid (EDTA) are used in combination to disinfect the root canal system and to eliminate debris and smear layers. An important concept that has emerged over recent years is the use of active physical methods for agitating these fluids to improve their penetration within areas that are not reached by endodontic instruments and to accelerate the chemical actions of these alkaline fluids against planktonic microorganisms, biofilms, soft tissue remnants and smear layers. Ultrasonic agitation and more recently pulsed lasers have emerged as two promising methods for activating endodontic irrigation fluids. Ultrasonic agitation with piezoelectric devices employs a moving tip, while laser agitation uses a stationary tip. Both methods cause cavitation, followed by implosions and shear forces which assist with debridement. Fluid streaming further enhances the activity of the fluids. While agitation enhances performance of irrigants, extrusion of fluids from the root canal during activation is a hazard that must be controlled.

## 1. Introduction

Effective chemo-mechanical instrumentation of the root canal system is crucial for the success of root canal treatment. Modern endodontic therapy involves the combination of the mechanical debridement of dentine with chemical agents for irrigation and disinfection, with the goal being the removal of all microorganisms from the root canal system. While existing instruments can shape the walls of root canal in a predictable manner, these can only contact part of the canal walls. As a consequence, soft tissue debris and microorganisms may present in both planktonic forms and in multilayered biofilms in parts of the root canal system [1].

An additional issue is that the use of hand operated or powered mechanical instruments produces a smear layer where instruments come into direct contact with the canal walls. This smear layer can contain microorganisms and their products and it may protect bacteria present within the dentinal tubules beneath it [2]. Removing this smear layer should enhance canal disinfection. Current methods of smear removal are not totally effective throughout the length of all canals, nor are they universally accepted. If the smear layer is to be removed, the method of choice seems to be the alternate use of alkaline solutions of ethylenediaminetetraacetic acid (EDTA), typically up to up to 17% concentration and sodium hypochlorite (NaOCl), typically up to 5% concentration [3]. The objective of this review is to describe physical methods for activating endodontic irrigation fluids to improve their chemical and physical actions and to explore the limitations of these approaches and the problems associated with fluid agitation, such as fluid extrusion beyond the confines of the root canal system.

## 2. Current Irrigation Protocols

In contemporary dental practice, a range of irrigation solutions are used in the root canal system of teeth during endodontics to remove soft tissue remnants, inhibit and physically remove microorganisms and dissolve smear layers created by instrumentation. In some cases, this means going beyond NaOCl and EDTA with additional cycles of irrigation. Adjuncts to NaOCl that provide additional disinfection include hydrogen peroxide (up to 3%), iodine potassium iodide (up to 5%) and chlorhexidine (up to 4%). Alternatives to NaOCl that have been examined for possible use in soft tissue dissolution include chlorine dioxide (up to 14%) and calcium hypochlorite (up to 10%). Alternatives to EDTA for smear layer removal the combination of 24% phosphoric acid and 10% citric acid, which has a pH less than 4 and chelators such as octenidine [4,5].

In most endodontic treatment protocols, EDTA is used as a ligand and chelating agent to sequester calcium ions and therefore remove smear layer. It can form a total of six bonds to calcium ions via its carboxylic and diamine subgroups. Solutions of the di-sodium salt of EDTA in water have a pH of at least 8.0, since solubility is low below this pH level. EDTA has low toxicity, as it remains in the extracellular fluid and is not taken up by human cells. The positive dental experience of the use of EDTA in endodontics parallels its positive track record for systemic use for heavy metal chelation therapy since the early 1950’s [6].

Sodium hypochlorite has a long history of use as an endodontic irrigant, dating back to 1936 when it was used both because of its germicidal properties and its ability to dissolve soft tissues of the dental pulp [7,8]. NaOCl only breaks down the organic component of smear layer [9,10] and so is used in conjunction with EDTA or another chelating agent.

The tissue dissolving capabilities of NaOCl are better at higher pH levels, with most commercial NaOCl solutions having pH values over 11. Hypochlorous acid (HOCl) is a weak acid, which dissociates to form hypochlorite ions (–OCl) and protons (H^+^) depending on the pH. Because HOCl is the active species for a germicidal action, antimicrobial efficacy is better when the pH is reduced to between pH 6 and 7.5 [11]. The concentration of –OCl is a key factor determining the soft tissue dissolution efficiency. The OCl− concentration is low when the pH is between 6 and 7.5 and under these conditions the tissue dissolution activity of NaOCl decreases markedly [12]. Thus, for any given NaOCl solution, there is an inherent trade-off in setting the optimal pH between the germicidal activity on the one hand and the tissue dissolution or cleaning activity on the other. 

## 3. Limitations of Conventional Treatments

The most widely used clinical technique of alternate irrigation with NaOCl and EDTA is effective in the removal of debris and smear layers in the coronal and middle level but the effectiveness in the apical third is much less [13]. This is largely due to the narrow dimensions of the root canal in this region and the physical problems of getting irrigant fluids to contact the root canal walls. Thus, when using NaOCl or EDTA solutions with conventional irrigation systems, it is common to find smear layers and organic debris in the apical third of the root canal system, compared to the coronal and middle third levels.

Irrigant solutions delivered with end-vented or side-vented needles into the root canals create fluid movement, which has a flushing action and can dislodge loosely bound debris and microorganisms from the walls of the root canal. The extent of the flushing action achieved when using syringe irrigation is very modest since irrigants often do not reach further than 1 mm from the needle tip [14]. The extent of flushing is influenced by the canal diameter, the canal cross-sectional shape, the depth of placement of the needle and the diameter of the needle.

Numerous problems arise when attempting to irrigate the root canal system including the formation of air bubbles and vapour locks, which prevent movement of fluid into the narrow confines of fins, isthmuses and lateral canals. This explains why no conventional instrumentation technique (hand or powered files), nor irrigating regimes appear capable of providing a completely clean canal [15,16]. Indeed, it has become widely accepted that the only effective way to clean complex areas of the root canal system, such as webs and fins, is through movement of the irrigation solution [17]. Adding to this, a 2012 Cochrane review concluded that there is no reliable evidence showing the superiority of any one individual irrigant used in endodontics [18].

## 4. Improvements to Irrigation Protocols

Much effort has been expended to improve the degree of contact of irrigating fluids by physical agitation of the fluid using mechanical vibration, ultrasonic energy or pulsed lasers. Physical agitation increases the contact between the fluids and the root canal walls and also increases the temperature of the fluids, which in turn enhances their chemical actions.

There has also been interest in following endodontic irrigation with laser photothermal or laser photodynamic disinfection, on the basis that these may inactivate any microorganisms that persist after irrigation routines have been completed [19].

As an adjunct to conventional chemo-mechanical debridement, photodynamic disinfection appears promising [20]. A key limitation to photodynamic disinfection is that the problems which affect conventional irrigation systems, such as vapour locks and limited penetration into the narrow confines of fins, isthmuses and lateral canals, also affect solutions which contain photosensitisers.

### 4.1. Improving Irrigant Flow in Root Canals Using Ultrasonic Agitation 

The effectiveness of irrigation relies on both the mechanical flushing action of the solution and the chemical ability of the solution to dissolve tissue [21]. For irrigating solutions to be effective, they must come into direct contact with the walls of the root canal. Hence, including appropriate surfactants can help achieve better wetting of these surfaces. This is particularly important when treating roots with small diameters, since irrigating solutions delivered using needles will have difficulty reaching the apical third of the root canal and solutions in this location are therefore less likely to benefit from agitation.

Shared characteristics of attempts to agitate fluids are that they can increase the temperature of the fluid, which can enhance their chemical and biological actions and can accelerate chemical reactions between agents in the fluid and the hard and soft tissues of the patient. Both sonic and ultrasonic approaches to fluid agitation have been employed, with varying levels of success.

A range of sonic endodontic instruments oscillate in the range of up to 6 kHz, with the Endo Activator (Dentsply Tulsa Dental, Tulsa, OK, USA) operating at 167 Hz. To be classified as an ultrasonic instrument, the vibrational frequency must exceed 20 kHz. Most dental ultrasonic scalers oscillate at frequencies between 25 and 30 kHz so can readily be adapted for agitation of NaOCl, EDTA, or other irrigant solutions [22].

Most modern ultrasonic handpieces are piezoelectric units, where the action of the tip is linear, creating a piston-like or tapping action, with nodes and antinodes along the instrument tip which sits within the irrigant solution. The solution can be placed into the canal and then agitated, or there can be continuous delivery of the solution into the canal. In both cases, the tip is held loosely and it does not touch the walls, so that the effect is passive.

Ultrasonic agitation generates a continuous movement of the irrigant and improves removal of debris by an effect termed acoustic streaming [23]. Ultrasonic activation of NaOCl can also improve the removal of a smear layer [24,25], however the primary goal is improved disinfection and improved removal of soft tissue remnants [26].

As well as acoustic streaming, ultrasonic energy creates cavitation at the tip of the instrument [27]. The consequential explosions and implosions produce shear stress, which can physically disrupt biofilms and can damage microorganisms. Current clinical protocols stress on using ultrasonic agitation with NaOCl. However, it is important to keep in mind that, ultrasonic agitation is not effective for enhancing the activity of EDTA [28].

### 4.2. Improving Irrigant Flow in Root Canals Using Middle Infrared Laser Activation 

The concept of laser treatment of the root canal in a dry state with no irrigant fluid present has been investigated thoroughly, with the Er:YAG laser (2940 nm wavelength) and the Er,Cr:YSGG laser (2780 nm wavelength) both able to remove debris and smear layers from root canal walls with a performance superior to other laser types, including the argon ion, carbon dioxide, Nd:YAG and near infrared diode lasers. All these lasers exert bactericidal effects but the Er:YAG and Er,Cr:YSGG lasers are most effective for hard tissue cutting, allowing them to directly ablate the walls of the root canal [29,30,31]. Such a direct ablative approach can only be considered as an adjunct to current chemical root canal disinfection protocols, since it cannot replace sodium hypochlorite [32].

Over the past decade, the use of laser energy to induce cavitation and acoustic streaming of intracanal irrigants has been investigated and several clinical protocols have been developed for fluid agitation using lasers. Laser activated irrigation (LAI) relies on the absorption of laser energy into water. Most work on LAI has used lasers operating in the middle infrared region, where the absorption of water is strongest, such as the Er:YAG laser (2940 nm wavelength) and the Er,Cr:YSGG laser (2780 nm wavelength) [33]. Absorption generates photoacoustic and photomechanical effects as steam and air bubbles are created in the irrigant. These bubbles form and then implode in the same way as those generated by ultrasonic instruments, with the major difference being that with lasers the tip position is stationary. Shockwaves that are generated from bubble collapse cause the fluid to move rapidly. The combination of shockwaves and fluid movement creates high shear stress on the root canal walls [34,35]. This can disrupt thick smear layers and remove debris from the walls of the root canal, greatly increasing the effectiveness of alkaline solutions of EDTA and ETDA with cetavlon (EDTAC) in removing smear layer [33].

In addition to EDTA and EDTAC, positive results have been reported for laser agitation of both NaOCl and QMiX solution (Dentsply Tulsa, Maillefer, Ballaigues, Switzerland). The latter is an irrigant containing EDTA, chlorhexidine and a detergent to lower the surface tension of the irrigant and enhance its penetration into biofilms [36,37].

Studies of the induction of cavitation bubbles in endodontic irrigants during LAI have documented high-speed fluid motion in the canal, despite the laser tip itself remaining in a stationary position [38]. This is an important clinical factor, since the operator does not have to manipulate the laser fibre to achieve the required activation of the irrigant fluid.

Several studies have reported LAI using NaOCl and have shown positive results in line with the benefits seen from passive ultrasonic activation. The overall effects are consistent with the concept that agitation can help address the challenge of residual biofilm in inaccessible parts of the root canal system [39,40].

Photon-induced photoacoustic streaming (PIPS) is a particular clinical protocol of LAI where Er:YAG lasers with conical tips are used to agitate an alkaline NaOCl irrigant to enhance debridement of the root canal system. The laser is used at a low pulse energy and the pulses have a short pulse duration (50 μsec) [41]. Cavitation induces fluid flow at rates about ten times higher than those seen from ultrasonic agitation, throughout the length of the canal [42]. This fluid flow enhances the removal of debris [43,44].

LAI undertaken using PIPS with NaOCl irrigants improves the penetration of the solution into dentinal tubules [45]. This technique has demonstrated good antimicrobial efficacy [46,47,48,49,50,51], as well as enhanced dissolution of pulpal soft tissues [52]. Nevertheless, there are mixed results regarding whether PIPS using NaOCl improves smear layer removal, with some positive studies [53,54] and others reporting minimal improvement [55,56].

A particular issue with PIPS is the extent of debris and NaOCl that can be extruded through the apex, when appropriate evacuation systems (such as negative apical pressure (EndoVac)), are not used simultaneously. More extrusion of material occurs using PIPS than when ultrasonic agitation is used [57,58,59,60].

### 4.3. Improving Irrigant Flow in Root Canals Using Near Infrared Laser Activation 

In addition to middle infrared lasers, there is interest in using near infrared diode lasers that emit at wavelengths of 940 and 980 nm, which are close to harmonics of the peak for water absorption. Both diode laser wavelengths have been shown to induce cavitation in water-based fluids, with the formation and implosion of bubbles of water vapour [61,62]. Peak laser power plays an important controlling role in inducing cavitation, making this process well suited to superpulsed diode lasers compared to those operating in chopped continuous wave mode. The generation of microbubbles from laser-initiated cavitation can be enhanced when EDTA, NaOCl or other water based solutions are supplemented with hydrogen peroxide to a final concentration of 3% [61]. 

Because of the high transmission of near infrared energy through dentine, diode laser irradiation of fluids can be accompanied by photothermal disinfecting actions in the dentine. Direct measurements of thermal changes when 940 and 980 nm diode lasers are used to generate cavitations in water-based fluids have shown only modest temperature changes on the external root surface, in the order of 4 degrees Celsius. Irrigation between laser exposures is highly effective in minimizing thermal changes on the root surface [62].

When 940 and 980 nm diode lasers are used for laser agitation of irrigant fluids, the temperature of the irrigant fluid within the root canal increases by up to 30 degrees Celsius [62]. This temperature elevation enhances the chemical actions of alkaline irrigant solutions such as 15% EDTA. Using a validated quantitative image analysis method to assess the removal of smear layer and debris [63], it has been shown that activating EDTAC with a 940-nm diode laser considerably improves smear layer removal, giving a superior result to the clinical ‘gold standard’ protocol using EDTAC alternately with NaOCl [64].

Recent studies of the absorption spectrum of water-based irrigant solution show that transmission and absorption results for NaOCl and EDTA are very similar to data for water, such that the active ingredients do not greatly alter the absorption qualities [65]. It has been suggested that when studying activation of root canal irrigants through LAI and employing cavitation at the tip of a laser fibre inside the root canal system as a method of agitation, an appropriate path length to consider is 1 mm, with the goal being less than 1 % transmission, so that here is a sufficient energy concentration to heat and vaporize a small volume of water [65]. 

### 4.4. Optical Tip Designs for Improved Laser Agitation of Irrigants

To enhance the directions of fluid motion induced by cavitation events, lateral emitting laser tips have been developed. A conical end can be formed on glass-based materials including those doped with fluoride, germanium or gallium using a tube etching process, to give enhanced lateral emissions. Such tips deliver more laser energy towards the root canal walls rather than towards the apex of the tooth. Such conical tips have proven superior to conventional plain-ended tips for removal of thick smear layers and debris [33].

Commercial optical fibres have been altered by a range of processes including tube etching with hydrofluoric acid, modified tube etching (after removing the protective polyimide coating), alumina abrasive particle beams, or by etching and particle beams used in various combinations. Improvements in conical tip design include micro-patterning of the surface using a specific method for etching, followed by particle beam abrasion and then additional etching. This creates a honeycomb-like series of circular facets for laser energy to exit the fibre, allowing a spherical emission pattern [66,67,68].

A honeycomb-like surface configuration of the tip, when used with Er:YAG and Er,Cr:YSGG lasers, improves lateral emissions (by 452 ± 69% and 443 ± 64%, respectively) and reduces the forward emissions (by 48 ± 5% and 49 ± 5%) when compared to conventional fibre tips. The ability to uniformly irradiate the walls of the canal using this modified tip design enhances treatment effects and prevents localized thermal damage to the root dentine and to the adjacent periodontal ligament. LAI of water-based irrigation fluids does not give clinically relevant temperature changes, allowing laterally emitting conical fibre tips to be used with irrigation fluids in the root canal without harmful thermal effects on the periodontal tissues [69].

## 5. Procedural Problems

### 5.1. Irrigant Fluid Extrusion

Because pulsed middle infrared lasers create both intense fluid movement as well as pressure waves in irrigant fluids within the root canal, there is a potential for irrigant fluids to be extruded from the apex into the adjacent periapical tissues. Studies that have quantified micro-droplet fluid extrusion beyond the apical constriction have compared Er:YAG and Er,Cr:YSGG lasers with both conventional and conical fibre tips positioned at distances of 5 or 10 mm from the apex. Most fluid motion with lasers occurred laterally. The volume of fluid extruded was found to be similar to that which occurred when conventional 25-gauge needles were used for canal irrigation. As would be expected, teeth with larger apical openings showed greater extrusion of fluid [70].

While both conventional plain ended fibres and conical tips create fluid movement largely in a forward direction, honeycomb tips generate agitation with fluid movement directed onto the walls, which lowers the risk of fluid extrusion beyond the apex [71].

### 5.2. Endpoints for Laser Fluid Agitation

When applying laser energy into irrigant fluids for LAI, it would be ideal if the clinician was able to assess the process of debridement as the treatment progresses, so that a defined endpoint can be reached and the treatment then stopped. Methods for real-time assessment of the microbial status of the root canal system have been developed, using laser fluorescence devices. Visible laser red light (wavelength 655 nm) elicits fluorescence emissions in the near-infrared range (790–840 nm) from the porphyrins present in bacteria. This can be used for detection of planktonic bacteria and bacterial biofilms [72], as well as for bacteria embedded within mineralized deposits [73]. As infection becomes established in the root canal system, there is a progressive increase in bacterial fluorescence readings over time. High fluorescence readings have been recorded in the root canals and pulp chambers of extracted teeth with radiographic evidence of periapical pathology and scanning electron microscopy evidence of bacterial infection. As endodontic treatment is undertaken, fluorescence readings reduce to reach the threshold level for "healthy" dentine. Using flexible optical fibres that penetrate into middle and apical thirds of the root canal, with either plain or conically modified ends, fluorescence assessment of the root canal has been undertaken successfully [74].

## 6. Methods for Assessing Effectiveness of Irrigation

A range of methods have been employed to assess cleaning of the root canal system, including scoring of debris removal [38,39,44], scanning electron microscopy with qualitative assessments of images [13,15,29,30,31] or digital image analysis [33,63,65], confocal microscopy [45] and micro-computed tomography [43]. Each technique provides specific insights into the effects created by the irrigant fluid. Thus, it is of value to employ more than one method when assessing the effectiveness of an improved irrigation approach. As the effectiveness of any given method may vary according to the region of the root canal system being examined, methods which allow the length of the entire root canal system to be inspected are preferred. As well as maintaining an emphasis on cleaning of the root canal, it is also important to monitor changes in the temperature of the root surface in order to gauge the possibility of thermal damage from warmed or agitated fluids [62].

## 7. Conclusions

The performance of alkaline solutions of NaOCl and EDTA in endodontics can be improved significantly when these are agitated using ultrasonic energy or pulsed lasers. These energy sources create fluid motion which improves the contact of the irrigant fluids with areas of the root canal walls that cannot be reached with rotary instruments. They also increase the temperature of the fluids, which in turn enhances their chemical actions on soft and hard tissues. 

An important aspect of any agitation protocol is that it should improve effectiveness but not introduce additional safety concerns. While EDTA has very low toxicity, NaOCl can cause significant irritation if extruded in large volumes into the periapical tissues.
